# What Role Can Process Mining Play in Recurrent Clinical Guidelines Issues? A Position Paper

**DOI:** 10.3390/ijerph17186616

**Published:** 2020-09-11

**Authors:** Roberto Gatta, Mauro Vallati, Carlos Fernandez-Llatas, Antonio Martinez-Millana, Stefania Orini, Lucia Sacchi, Jacopo Lenkowicz, Mar Marcos, Jorge Munoz-Gama, Michel A. Cuendet, Berardino de Bari, Luis Marco-Ruiz, Alessandro Stefanini, Zoe Valero-Ramon, Olivier Michielin, Tomas Lapinskas, Antanas Montvila, Niels Martin, Erica Tavazzi, Maurizio Castellano

**Affiliations:** 1 Dipartimento di Scienze Cliniche e Sperimentali dell’Università degli Studi di Brescia, 25128 Brescia, Italy; maurizio.castellano@unibs.it; 2 School of Computing and Engineering, University of Huddersfield, Huddersfield HD13DH, UK; m.vallati@hud.ac.uk; 3 PM4Health-SABIEN-ITACA, Universitat Politècnica de València, 46022 València, Spain; cfllatas@itaca.upv.es (C.F.-L.); anmarmil@itaca.upv.es (A.M.-M.); zoevara@itaca.upv.es (Z.V.-R.); 4 Department of Clinical Sciences, Intervention and Technology (CLINTEC), Karolinska Institutet, 171 77 Stockholm, Sweden; 5 Alzheimer Operative Unit, IRCCS Istituto Centro San Giovanni di Dio Fatebenefratelli, 25128 Brescia, Italy; steorini@gmail.com; 6 Department of Electrical, Computer and Biomedical Engineering, Università di Pavia, 27100 Pavia, Italy; lucia.sacchi@unipv.it; 7 Fondazione Policlinico Universitario A. Gemelli IRCCS, 00168 Roma, Italy; jacopo.lenkowicz@unicatt.it; 8 Department of Computer Engineering and Science, Universitat Jaume I, 12071 Castelló de la Plana, Spain; mar.marcos@uji.es; 9 Human & Process Research Lab (HAPLAB), Department of Computer Science, School of Engineering, Pontificia Universidad Católica de Chile, 3580000 Santiago, Chile; jmun@uc.cl; 10 Department of Oncology, University Hospital of Lausanne, 1011 Lausanne, Switzerland; Michel.Cuendet@chuv.ch (M.C.); Olivier.Michielin@chuv.ch (O.M.); erica.tavazzi@phd.unipd.it (E.T.); 11 Swiss Institute of Bioinformatics, UNIL Sorge, 1015 Lausanne, Switzerland; 12 Radiation Oncology, Réseau Hospitalier Neuchâtelois, 2000 La Chaux-de-Fonds, Switzerland; berardino.debari@rhne.ch; 13 Department of Oncology, Lausanne University Hospital, University of Lausanne, 1015 Lausanne, Switzerland; 14 Norwegian Centre for E-health Research, University Hospital of North Norway, 7439 Tromsø, Norway; Luis.Marco.Ruiz@ehealthresearch.no; 15 Dipartimento di Ingegneria dell’energia dei sistemi del territorio e delle costruzioni, Università degli Studi di Pisa, 56126 Pisa, Italy; a.stefanini88@gmail.com; 16 Department of Cardiology, Medical Academy, Lithuanian University of Health Sciences, 44307 Kaunas, Lithuania; Tomas.Lapinskas@kaunoklinikos.lt; 17 Department of Radiology, Medical Academy, Lithuanian University of Health Sciences, 44307 Kaunas, Lithuania; montvila.antanas@gmail.com; 18 Data Analytics Laboratory, Vrije Universiteit Brussel, 1050 Ixelles, Belgium; niels.martin@uhasselt.be; 19 Research Foundation Flanders (FWO), 1000 Brussel, Belgium; 20 Hasselt University, 3500 Hasselt, Belgium; 21 Department of Information Engineering, Università degli Studi di Padova, 35122 Padova, Italy

**Keywords:** clinical guidelines, process mining, healthcare

## Abstract

In the age of Evidence-Based Medicine, Clinical Guidelines (CGs) are recognized to be an indispensable tool to support physicians in their daily clinical practice. Medical Informatics is expected to play a relevant role in facilitating diffusion and adoption of CGs. However, the past pioneering approaches, often fragmented in many disciplines, did not lead to solutions that are actually exploited in hospitals. Process Mining for Healthcare (PM4HC) is an emerging discipline gaining the interest of healthcare experts, and seems able to deal with many important issues in representing CGs. In this position paper, we briefly describe the story and the state-of-the-art of CGs, and the efforts and results of the past approaches of medical informatics. Then, we describe PM4HC, and we answer questions like how can PM4HC cope with this challenge? Which role does PM4HC play and which rules should be employed for the PM4HC scientific community?

## 1. Introduction

New evidence and discoveries in medicine are continuously pushing forward the amount of information that medical professionals need to process to keep their knowledge compliant to the standards required for research activity and clinical practice. Drugs, diseases, bio-markers, or equipment are all instances of these ever improving assets inherent to a physicians professional knowledge base, which are incorporated into Clinical Guidelines (CGs) for standardized and controlled use. Consequently, CGs are frequently updated at national or international level to keep the processes aligned with the best practices and the latest scientific evidences. Further, CGs can be also defined and modified at the hospital level to get a reasonable trade-off between the optimal outcome and the available resources. In this scenario, CGs play a pivotal role for the real-time assessment of patients’ care pathways, given the growing interconnection of data sources both at the hospital level and between hospitals. A key concept here is the Evidence-Based Medicine (EBM) paradigm [1], namely, the comparison between what happens in the practice and what it is suggested from the data. To be effective, EBM relies on an interconnection between the physicians’ knowledge base—protocols, CGs, and consensus—and knowledge gathered from different data sources through proper IT infrastructures and implementation strategies.

The relation between CGs and computers is not a new topic in medical informatics; the so-called Computer Interpretable Clinical Guidelines (CIGs) were proposed in the 1990s (see, e.g., in [2]) to try and address this challenge of knowledge transfer between humans and computers. After an initial flourishing of formalisms and languages [3], we now have to face the fact that the actual applications of CIGs are limited. The reasons for this are heterogeneous, but seem to be mostly related to clinicians’ limited understanding and trust in the underlying models, thus creating poor engagement in their usage [4,5,6].

The last decade saw the rise of a field of study, Process Mining (PM), which addresses the problem of monitoring and improving actual processes execution by extracting knowledge from event logs stored in IT systems and data warehouses. The kind of techniques, methods, and tools used in PM are often referred to as a *bridge between data mining and Business Process Management (BPM)* [7,8].

Broadly speaking, a simple description of PM can be made from its three conceptual building blocks: Process Discovery (PD), Conformance Checking (CC), and Process Enhancement (PE). Given the input data in the form of an Event Log, PD algorithmically represents the underlying processes, CC compares them to a predefined process model, and PE returns a process model with an increased fitting with respect to a given Event Log. PM was initially proposed as a general purpose analytical framework, with several domains of application such as manufacturing, finance, retail, and customer service. Nevertheless, the healthcare sector was soon recognized as a remarkable possible extension of the PM scope [9], in particular to identify bottlenecks and improve hospital performances through PD, and to provide an evidence-based comparison between expected and actual processes execution through CC. Indeed, in the last few years there has been a rising exploration of PM applications in healthcare, leading to the definition of a new discipline: Process Mining for Healthcare (PM4HC), whose high-level purpose is to create a unique discipline able to satisfy the requests coming from the healthcare workers and institution (http://www.processmining4healthcare.org/), MOOC (https://www.futurelearn.com/courses/process-mining-healthcare), and alliances (http://pods4h.com/).

The group behind this paper, composed of hospitals and research centers, proposes a vision of the role of PM4HC which aims to address questions such as the role played by CC analysis in comparison with the well-established but underexploited field of CIGCs, or the implications of PD in a context where the actual process execution can diverge from the mere CG expectation due to “exogenous” or hospital-related factors like the availability of research protocols, differences in equipment, and resource or staff constraints. Discussion will always aim to bring PM4HC analyses closer to the clinicians needs, and to make PM4HC more effective and usable in the clinicians’ daily practice.

The paper is organized as follows. First, the reader is introduced to the complexity of data collection in medical informatics; this allows us to define terminology and describe the big picture for data extraction, Event Log building, and CIGs representation. Then, for the sake of clarity, emphasis is put on the genesis of the idea of CG, and the different meanings this term carries within itself. Then, CIGs are described, to give context to the most relevant developments in CGs research. The overlap between CICG and BPM is also addressed, and how CG and PM have evolved over the last years. Finally, challenges and opportunities for PM4HC with respect to CIGs are discussed.

## 2. The Data: The Starting Point

In this section, we present the most common health data sources and infrastructure with the aim of providing an overview of the wide range of acronyms exploited in data collection and management. When measuring the adherence with CGs, researchers often have to cope with heterogeneous data sources. Making sense of disparate data sets involves the harmonization at a syntactic and a semantic level. Syntactic harmonization involves the representation of data using common normalized schemes. Semantic harmonization concerns the binding of the entities that conform the harmonized schema to a standard terminology in order to to accurately define their semantics. These combined activities are usually the most expensive stage needed before performing PM. On top of that, there is a plethora of different “standards” for the representation of data schemes and many terminologies for the representation of its semantics that need to be considered, and they greatly vary even between sub-topics of healthcare. Needless to say, the abundance of standards is a major issue to face. However, some (fortunate) investigations only require data from one or few different data sources. Even under such circumstances, it is not uncommon to have to cope with a kaleidoscope of repositories built in different times, for different aims, by different companies.

The next subsections provide a more detailed picture of the different aspects and elements that have to be faced when dealing with data sources in the health domain.

### 2.1. Data Sources

In a hospital, the most common data sources are Research Electronic Data Capture (REDC) and Electronic Health Records (EHR): the former are commonly aimed at supporting research, while the latter are more generally oriented at supporting daily activities. EHRs are often composed of various subsystems, such as Radiology Information System (RIS) and Laboratory Information System (LIS). Another data source is Hospital Information Systems, that are devoted to acquire, store, and resume the administrative events that occur in the institution.

When collecting structured data from these sources, the most common issues often concern the adoption of different information standards (e.g., HL7 CDA, HL7 FHIR, openEHR, etc.) and terminologies for representing clinical procedures, drugs, adverse events, diseases, LAB exams, etc. (e.g., LOINC [10], SNOMED [11], ICD [12], and CTCAE [13]). However, even if standards exist, they are often implemented partially, badly, or completely ignored, and proprietary solutions are still quite common.

### 2.2. Giving Meaning to Data

One of the biggest challenges to apply PM algorithms, as well as other Artificial Intelligence (AI) techniques, is data quality [14]. EHR data representation formats are often oriented towards data readability and interpretation by humans. Free text sections, ad hoc abbreviations, and local medical jargon are common in clinical notes. However, AI algorithms require high-quality data, particularly in the sense of structured data with very clear semantics, in order to allow for its correct interpretation outside their original context and across organizational boundaries. To that end, different types of semantics need to be carefully represented. Several possible classifications exist [15,16,17]. In the following, we provide a minimal division leveraging on proposed views.

- Data semantics are concerned about the content and structure of observations over a biological subject or organization. For example, the data structure for representing the items contained in a urine analysis (color, pH, specific gravity, presence of nitrites, glucose measurement, etc.).
- Contextual semantics are needed to correctly interpret data semantics. They concern contextual aspects such as times of events, display used for a specific measurement, performer of an activity, and place where an event occurred. They are often tightly linked to data semantics in most of the standards for representing clinical information.
- Workflow semantics concern the specification of the order of biomedical events. These semantics are needed to understand temporal, conditional, and causal relationships among various events. Examples include which event occurred before a nosocomial infection, which activity is executed after detecting stroke in the emergency ward, and what order should preoperative activities follow. These kinds of semantics are represented by workflow specification standards such as the openEHR Task Model, GLIF, or BPM.

Languages have been developed for representing and computing each type of semantics. Regarding data and contextual semantics, they are often represented in the same computational artifact as they are tightly linked. In the case of openEHR, the Archetype Definition Language (ADL) is used to define information schemes that model the medical journal parts. In HL7 CDA, XML is mostly used to specify the clinical documents that are shared across health providers. In the case of HL7 FHIR, both XML and JSON are used to define clinical and administrative structured extracts (resources and profiles in the HL7 jargon) that are exchanged across health information systems. These artifacts are generally known as Clinical Information Models (CIMs) or Detailed Clinical Models (DCMs) and provide a syntactically harmonized view of the information represented. In order to precisely define their semantics, CIMs allow for binding their items to biomedical terminologies. In this way, a code or URI references a concept in a standard terminology that provides a description for the semantics that the information item represents. The terminology is sometimes expressed using logic-based languages such as the Web Ontology Language (OWL). This family of languages is suitable for maintaining and reasoning about complex models with thousands of classes and relationships such as SNOMED-CT (https://confluence.ihtsdotools.org/display/DOCOWL/SNOMED+CT+OWL+Guide). However, they are not optimal for representing CIMs as the representation of data constraints leads to tractability problems [15,18]. In CDSS, CIMs are often simplified to only represent a subset of the full EHR with the information that is useful for decision support algorithms. This subset is called Virtual Medical Record (VMR). VMRs can be implemented as a persistent repository [19] or able to dynamically create a EHR view with a query language (e.g., AQL) [20].

For workflow semantics, several languages have also been proposed. In openEHR, the openEHR Task Plan uses ADL for specifying workflows [21], another language for CDS rules is the Guideline Definition Language developed by Chen et al. [22,23]. In the CIGs realm, workflows have been traditionally represented using two main alternative approaches. On the one hand, many workflows can be specified using syntactic standards that allow for rich data constraints definitions (e.g., XML). On the other hand, some workflow models have used logic-based languages (OWL) for describing their semantics [24]. The latter have more expressive power and facilitate the reuse of biomedical ontologies. However, if the process model is not designed with attention to computational features, it may lead to an intractable, or even undecidable models.

## 3. Clinical Guidelines

This section provides an overview of Clinical Guidelines (CGs) and of the wider field of structuring medical knowledge.

### 3.1. Definition and History of CGs

In a field where, historically, clinical practice was seen as the “art of medicine”, the need for structuring physicians’ knowledge and experience emerged already in remote times: the first known formal clinical guidelines may have been written by Hippocrates in the Hippocratic Corpus, where he summarized medical knowledge and prescribed practices for physicians. In the modern age, the most famous CG appeared in 1753 when James Lind paved the way to clinical trials on human subjects with his famous study on scurvy in the British navy, documenting the collected evidences in his treatise [25]. Starting from that moment, over the following the decades, CGs became more popular but medicine still remained balanced between being an art and a science, with no structured approach in sharing the clinical knowledge.

In contemporary history, a big step ahead came in 1962 with the approval of the US Food and Drug Administration Kefauver–Harris Act, in which a rigorous empirical testing for clinical trials on human beings was legally required to establish claims on drug efficacy [26]. However, the bases to understand the importance of the CGs were posed in the 1970s and 1980s, when Sackett, Eddy, and Cochrane proposed initial evidential rules for guiding clinical decisions [27,28,29]. Against this background, with the introduction of Evidence-Based Medicine (EBM) in 1991, Guyatt [30] opened the doors to well-defined and structured strategies like tracking down publications directly relevant to the clinical problem at hand, critically appraising these studies, and applying the results of the best ones. He also explicitly outlined how to apply the scientific method in determining the optimal management of the individual patient [31]. This new approach to medical practice grew over the following years, during which Guyatt collaborated with many academics to form an international working group, the Evidence-Based Medicine Working Group [1]. In 1993, Chalmers founded the Cochrane Collaboration with the mission to promote evidence-informed health decision-making by producing high-quality, relevant, accessible systematic reviews and other synthesized research evidence [32].

In this context, the modern age of the clinical guidelines arose. In 1990s, in its report [33] the Institute of Medicine (IOM (http://www.iom.edu.np/)) gave the formal definition of clinical guidelines as “systematically developed statements to assist practitioner and patient decisions about appropriate healthcare for specific clinical circumstances” or “statements that include recommendations, intended to optimize patient care, that are informed by a systematic review of evidence and an assessment of the benefits and harms of alternative care options” [34]. These concepts were extended over the following years, and in 1999 CGs were described as aimed at “improving the quality of patient care, by encouraging interventions of proven benefits and discouraging the use of ineffective or potentially harmful interventions, to reduce unnecessary variations in practice, to lessen disparities, to empower patients, and to influence public policy” [35].

Even though the general idea of CGs was established at the end of the last century, the real-world exploitation needed further refinement: even if a CG represents the collection of the known scientific literature at a certain point, not all the proposed actions are supported by the same extent in literature. CGs were therefore flanked by the “level of evidence“ of the statements, a pivotal concept in the clinical choice forming a strong basis when a physician is called to balance between his/her own experience and what is suggested in a CG. For that reason, in 2003, the Grading of Recommendations Assessment, Development and Evaluation (GRADE) group developed the GRADE approach [36], which became the gold standard for study evaluation [37] and is currently adopted by the WHO when developing recommendations [38]. Plenty of different scales for assessing CGs attributes, among which the level of evidence, have been then proposed over time by different institutions, such as the Oxford Centre for Evidence-based Medicine (https://www.cebm.net/2016/05/ocebm-levels-of-evidence/), the National Academy of Medicine [34] and the National Institute for Health and Clinical Excellence in the United Kingdom [39] in 2011, the Guidelines International Network in 2012 [40], and the World Health Organization in 2014 [38].

**Figure 1:**
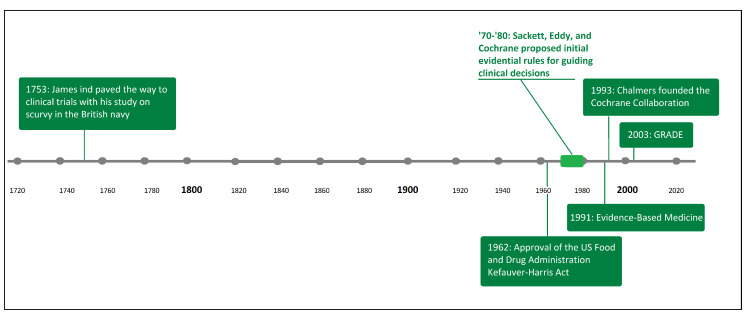
A general overview of some of the most relevant milestones in the modern age of Clinical Guidelines.

An overview of the most relevant milestones of CGs in the modern age is provided in Figure 1.

### 3.2. Between the Shades of CGs

What is commonly referred to as CGs in the medical informatics domain can stand for different classes of indications in medicine.

For example, the WHO defines the following four types of CGs [38],

- the *full guidelines*, that provide complete coverage of a health topic/disease, include recommendations in relation to all aspects of the topic (e.g., surveillance, diagnosis, public health, and clinical interventions), and need to be fully based on systematic reviews of the evidence for each aspect;
- the *standard guidelines*, that are produced in response to a request for guidance in relation to a change in practice or controversy in a single clinical or policy area, and that are supported by systematic reviews of the evidence, but not expected to cover the full scope of the condition or public health problem;
- the *rapid advice guidelines*, that are produced in response to a public health emergency and, for this reason, are mainly only evidence-informed and may not be supported by full reviews of the evidence; and
- the *compilations of guidelines*, that contain current recommendations from WHO and other sources, but does not include any new recommendations.

Moreover, in order to address the need of standardization and communication of the clinical practice at further implementation levels, more evidence-based tools were developed beside the CGs:

- The clinical consensus statements are a collection of opinions on a particular aspect of medical knowledge, generally agreed by a representative group of experts in that area upon an evidence-based, state-of-the-art knowledge. In contrast to CGs, which are based primarily on high-level evidence, clinical consensus statements are more applicable to situations where evidence is limited or lacking, yet there are still opportunities to reduce uncertainty and improve quality of care [41]. Moreover, the consensus statements synthesize new information that may have implications for revaluation of routine medical practices, and they do not provide specific algorithms or guidelines for practice because these depend on cost, available expertise and technology, and local practice circumstances [42].
- The clinical protocols are basically rules of how to proceed in a certain situation and consist of a set of criteria outlining the management steps for the specific clinical condition [43,44]. Because of their higher level of detail, they can be seen as more specific than guidelines.
- After their introduction in the practice in 2008 [45], clinical checklists are experiencing a wide spread [46]. Structured as a schematic list of actions or controls, checklists are inserted into various points of the clinical process, ensuring that providers do not forget crucial steps during either routine, mundane tasks or dynamic, emergent events [47].

However, the aforementioned definitions are not fully recognized and adopted by all the institutions (hospitals, government agencies, etc.) and small changes are commonly admitted for specific issues. The UK National Health Service (NHS), for example, also introduced the term *Guidance* for the following reasons. “*The term guidance is used, rather than guideline, in part because this is the term adopted by NICE, and in part because guideline is often used as an umbrella term for guidance, clinical protocol and care pathway*” (http://www.openclinical.org/docs/ext/briefingpapers/bensonPathways.pdf).

Even if the differences between CGs and Clinical Protocols, for example, can be relevant from the clinical point of view they are often irrelevant from the perspective of their formal (technical) representation. This is because differences are often not in the processes or in the workflows they describe, but in the legal implications and the way they should be put in practice in the daily clinical activity. For this reason, in the rest of the paper we will use the term CG to refer to all the aforementioned variations which can be represented with the common tools and methods adopted for representing CGs.

### 3.3. Guidelines in Health Management

Despite the fact that CGs were originally developed to support clinical decision-making, nowadays they are being used for broader purposes such as institutional policy, to inform insurance coverage, for deriving quality of care criteria, and for medico-legal liability standards [48].

Specifically, CGs may strongly affect the management of healthcare services, with very impactful effects on health process management and resource use [49,50,51,52]. The positive effects of CGs on the effectiveness of care treatments by supporting the clinical decision-making, are widely shown in the scientific literature, and most of the medical staff currently recognize the potential of CGs in medical practice [53]. However, doctors tend to ignore the potential positive impacts on efficiency that clinical guidelines can entail, being skeptical about their application for this goal. This is related to the broader problem of the medical staff’s limited inclination to take on a managerial mindset, neglecting that health treatments are not free and that the budgets of healthcare programs are limited.

Nevertheless, scientific literature proposes many ways in which CGs may be exploited for improving the healthcare service management, providing also some empirical evidences. Specifically, the potential applications conceived seem to be at least the following.

- CGs may avoid unnecessary diagnostic tests, which are routinely performed in daily practice in hospitals, helping doctors to select the most suitable ones based on predefined procedural indications. This may permit increased hospital efficiency, avoiding wasting resources on unnecessary testing, while maintaining the level of care provided to patients [49,54]. In addition, a reduction in the number of test requests also leads to an improvement in the service level and care effectiveness because it allows to cut the waiting lists (queues) for the tests and therefore to reduce the waiting times for patients who really need them [51,55]. This rationale, explained for diagnostic tests, can be extended also to medical treatments although probably to a lesser extent.
- CGs establish a benchmark to periodically evaluate the care paths of patients affected by a specific disease [52,56]. This makes it possible to match the activities which were actually executed against CGs. Therefore, it permits providing detailed feedback on the decisions of medical staff and/or on the unit management according to the different specific diseases. Such indications may allow doctors to identify their evaluation errors and to improve their future attitude.
- CGs may help health managers in resource planning, especially when a new center/unit must be set up [57]. Indeed, CGs may be exploited to estimate the activities needed for an “average patient” admitted with a certain disease.
- CGs can be exploited by insurance companies or health authorities to analyze whether hospitals are, for a particular patient group, complying with CGs when executing their diagnostic and treatments activities [58,59]. Non-compliant behavior, if large and economically advantageous, may be linked to abuses or mistakes.

Given the strong increase in the health service demand and, at the same time, the decrease in the resources available for healthcare systems, healthcare managers are fascinated by the possibility of applying CGs to optimize the use of resources and to increase the care service level. Unfortunately, although there is some empirical evidence, the real applications of CGs to improve the health process management are quite scarce.

## 4. Computer Interpretable Clinical Guidelines

**Figure 2:**
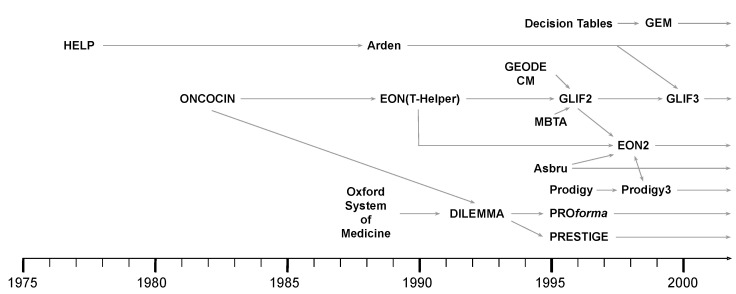
The first era of Computer Interpretable Clinical Guidelines (CIGs). Adapted from the work in [69].

Starting from the idea that accepted evidence-based guidelines, if implemented, could substantially reduce inappropriate variation in the healthcare practice, the scientific community proposed approaches to formally represent CGs and clinical procedures “*to develop CIG-based decision support systems (CDSSs), which have a better chance of impacting clinician behavior than narrative guidelines*” [3]. The first attempts to face this challenge were made with ONCOCIN [60] and HELP [61] at the beginning of the 1980s, but it is with the widespread adoption of EHRs that CIGs emerged as a relevant field of research [3]. Starting from the late 1990s, the scientific community directed its attention towards this area, and developed many languages for the representation and tools for the execution of CIGs, such as Asbru [62], GLIF [63], GLARE [64], PROforma [65], EON [66], GUIDE [67], Decision Tables [68], and many others. Figure 2 visually recaps the first era of such languages, as shown in [69].

The most famous comparison of CIG models was presented in 2003 [70]. By considering seven languages (GLIF, EON, Asbru, PROforma, Prodigy, Prestige, and GEM), it showed that, even in the presence of differences in the constructs made available by each language, all of them were capable to represent CGs as a set of actions (the so-called *plan*) that are executed over time. The general architectures were also similar, and consisted of a language and an execution engine, with the possibility to investigate nested processes and explicitly manage temporal constraints. The languages also had to cope with similar challenges: for example, although some of them (e.g., GLIF and Arden Syntax) were designed for reusing procedural knowledge across organizations, the most relevant issues related to the adaptation to local contexts and the connection with the EHRs.

The *golden age* of the aforementioned cooperative (and also competitive) experiences persisted for a few years. The mature recap of Sonnenberg and Hagerty [71] describes that (i) there was no dominant CIG system, and (ii) the major challenge in the development of interoperable CIGs was the agreement on a standard vocabulary, in particular in linking the CIGs to EHRs to actually make the CIGs widely usable in the daily clinical practice. This missing connection between CIGs and EHR limited their broad adoption [3,72] and is recognized as a common challenge by all computerized CDSS [4,72].

Despite the fact that many of the aforementioned languages and models are no more in the spotlight, others obtained remarkable visibility: the Arden Syntax [2], one of the oldest, was born in the 1990s and integrated in HL7 and ANSI in, respectively, 1999 and 2014. It is still on the open scene, with structural changes being proposed: for example, to include user-defined functions within the Medical Logic Module (MLMs) [73], to embed expressions into strings [74] (published in 2018 and 2019 respectively), or to harmonize it in the ecosystem of more recent and promising standards [75].

### Linking CIGs to Data

An original approach to connect CIGs to data in a flexible and vendor-independent way was the GELLO language [76]. Based on the Object Constraint Language (OCL), GELLO was an object-oriented language for expressing logical conditions and computations in GLIF3 and its object-oriented model is designed to fit with the object-oriented HL7 RIM (a reference information model split in area such as *Entities*, *Roles*, *Participations*, and *Acts* for framing the semantic of the HL7 messages).

The idea that CIGs systems (and more in general CDSS) could look at the data via an abstract representation led to the idea of a Virtual Medical Record (VMR), which was a goal of many important initiatives (such as [77] or the HL7 VMR). The first CDSS/CIG approaches following this idea use either an HL7-based VMR [78,79] or an openEHR archetype-based one [80].

On this track, the Guideline Definition Language (GDL) [22] is a rule-based language that allows to directly reference openEHR archetypes and allows the seamless integration of CDSS modules with openEHR-based EHRs [23]. Both GDL and the openEHR Task Planning models are designed to run over openEHR compliant repositories and when the EHR contains data stored in format different from openEHR, a preprocessing stage can be performed for making it openEHR compliant [81].

Recently, CSL [82] has been introduced as a paradigm aimed at bridging the gap between EHRs and CDSSs: it implements a sort of Object-Relational Mapping and allows CDSS to query the EHR in real-time by exploiting an abstract high-level language. By design it is meant to be simple, data oriented, customizable, and does not provide a mapping to a known ontology (the user is is not constrained to any specific information model). Even if it is mainly devoted to fill the gap between CDSS and EHR, it gave proof to be usable for representing CGs.

## 5. CIGs and BPM

Over the years, the CIG field has proposed languages with a wide range of constructs adapted to the variety and heterogeneity of CG knowledge. Peleg et al. identified two main dimensions related, respectively, to the structuring of CG procedures in plans of decisions and actions, and to the link with patient data and medical concepts [70]. Regarding the former dimension, similarities between the field of CIGs, on the one hand, and the domain of BPM and workflow management, on the other hand, have been exploited for over a decade. In this line of work, some approaches have sought the integration of CIGs into workflow management systems (WfMSs) [83], while others have focused on the application of BPM to the representation of CGs.

Early advocates from the CIG field of the integration with WfMSs are Stefanelli et al. [84,85]. They propose a variant of the workflow concept called *careflow* to support clinical practice by modeling CGs in the context of workflow, communication, and resource management services [86]. Other authors have also investigated the implementation of CGs in specific healthcare environments, taking into account resources and organizational settings [87,88,89].

The work by Mulyar et al. is one of the first of approaches applying BPM and workflow results [90] to the CIG field. They analyze a subset of CIG languages on the basis of the implementability of workflow (control flow) patterns in these languages. Another example is a formal method to assess this implementability, using PROforma as an illustration [91]. Closely related, a different work limits the scope of the implementability analysis to the workflow patterns that can be found in CG texts [92]. While the authors of [90] conclude that CIG languages have major limitations because they do not support the whole set of workflow patterns, the authors of [92], focusing on the patterns that are relevant in the context of CGs, conclude that CIG languages are very well suited for describing them. Besides workflow patterns, the use of the BPMN notation has been proposed as an intermediate step to simplify the acquisition of CG procedural knowledge in terms of CIG languages, arguing that BPMN can be easier to comprehend and handle for clinicians [93].

Against this background, the benefits of an extensive cross-fertilization of ideas and technologies between the CIG and BPM fields are yet to come. On the one hand, due to the adaptability and flexibility needs specific to clinical processes, the healthcare sector has been one of the last ones to adopt BPM technologies [94]. On the other hand, mature/consolidated proposals from the CIG field have been disregarded in the BPM field until quite recently, notably the explicit modeling of decision-making policies (see, for example, in [95]). As a result, the joint application of CIG and BPM technologies, providing an effective support for hospitals covering both clinical decision-making and managerial aspects, has not been achieved.

It has been argued that CIG and BPM systems used independently do not address important aspects of clinical processes [94]. It has even been questioned whether it is possible (or adequate) to develop a comprehensive framework integrating all CIG aspects, including design, modeling, formalization, integration into workflow, etc. [6]. In this situation, there is a growing consensus that the most promising way forward will be to promote and harmonize the use of the most appropriate frameworks for each of these aspects.

## 6. PM for Healthcare: The Perspectives on CGs

Even if PM is designed to be general purpose, healthcare represents one of its most promising application domains, as suggested by a recent review of the area [9] based on 1278 articles. Current researches acknowledge that PM4HC poses specific, non-trivial challenges, as hospitals do not work as factories and the patient care pathway cannot be considered as if produced by a conveyor belt system, as correctly reported in [96,97]. Indeed, the care pathway of a patient is often a long and demanding journey, of which the complexity is directly dependent on the high number of professionals with different expertise, diagnostic opportunities, and therapeutic schemes that are available for each specific clinical case. The care process often involves multidisciplinary teams in order to choose the best possible approach among several treatment options, based on a variety of evidence such as laboratory tests, imaging data, and medical visits. Moreover, patients can also play a role themselves, as they might make the process deviate from the expectations according to actions or denials based on their values, beliefs, and also fears. Even if the domain has well-established strategies, addressing this complexity implies that each single task—treatment or diagnostic procedure—can be thought of as a chess move, where the physician and the patient wait to see the results, before deciding the next move. In a nutshell, to appropriately apply PM4HC, the analyst must reckon that medical treatment processes are highly dynamic, highly complex, increasingly multidisciplinary, and often ad hoc [98]. Therefore, the dedicated field of PM4HC is being developed to calibrate general PM on the needs of the clinical application domain.

According to the experience of these first years of PM4HC, what physicians are mainly and foremost keen on is monitoring how patients flow through the graph of CGs, in order to check its conformance and easily identify those groups of patients that did not follow it, in the quest of understanding why that was the case and the related implications. In a pioneering paper, Mans et al. [99] analyzed four typical questions from medical process specialists, mentioning *Do we comply with internal and external guidelines?* In 2015, the same authors [100] represented a Clinical Guideline with DECLARE [101] and performed CC with ProM [102]. Later, in 2016, a review [103] highlights how CC to a predetermined model (not automatically mined) has been applied in 14 of the 71 considered studies. A more specific study for oncology [104] counts 7/37 papers where PM4HC was adopted in measuring the compliance on CG. More recent papers concerning the application of CC with CGs are found in [105] for alcoholism, in [106] for the treatment of skin (melanoma) cancer, and in [107,108] for colon and rectal cancers.

Another relevant point to investigate concerns the patients’ individual differences in diagnostic/treatment care. Individual differences cause great variances in the execution of the healthcare processes. Moreover, diseases are not static: they evolve over time in different directions. PM4HC can construct individual behavior models [109], including not only the individual preferences, and social, mental, and health determinants, but also the variability and evolution of diseases over time. These individual models can be compared, and using PM4HC, the differences between the standard CG and the individual behavior model for each patient can be measured. This allows to not only provide a way to measure the adherence of the patient treatment to the CG, but also to analyze in which part of the process there are problems. Moreover, these results can be presented in a human-understandable way to health experts. This permits developing the integration between the medical evidence and experts’ running knowledge traditionally desired in EBM paradigm.

The analysis of the deviations can also be extended from a single-patient analysis to a set of patients: in this case physicians’ involvement is crucial for understanding what is actually happening in those groups, and why. PM4HC produce human-understandable models allowing the extraction of evidence using a data driving paradigm permitting the application of interactive solutions [110]. This could be a powerful solution to support physicians and other healthcare professionals in the comprehension of patients’ pathways [111]. Moreover, for patients outside the standard CG, these models could be very valuable for interpreting the patient circuits in a CG, stratify them into groups with clinical meaning. These groups represent sets of subpopulations with similar characteristics.

In this line of work, there is increasing interest in discovering more accurate stratification groups that permit a better understanding of the clinical cases, and therefore may allow a better care delivery and maximize the process value based on each group conditions [112] and PM4HC in combination with Trace Clustering techniques could tackle this issue. Clustering algorithms are unsupervised data mining solutions that are able to group traces that have similar behavior, maximizing differences with the rest of groups [113].

Nowadays, the growing interest of physicians and computer scientists in PM can enable PM4HC to become a fertile ground to collect the needs, visions, and efforts of a multidisciplinary community in delivering prototypes and products for the daily clinical practice.

## 7. Conclusions

Evidence-Based Medicine is a commonly recognized approach to share knowledge and cope with the new findings in medicine. The expected benefits range from an increased and more homogeneous quality of care to a better governance of the Public Healthcare Systems. In order to suggest procedures and therapies for diagnosis or treatments, this paradigm exploits CG, Consensuses, Protocols, etc., which are largely used in the daily practice, but rarely integrated in Decision Support Systems. For this reason, medical informatics was expected to give a relevant contribution in collecting, storing, distributing, and supporting the decisions of the healthcare workers in their clinical activities. In fact, some proposals for dealing with the mentioned aspects can be found in different areas of medical informatics, such as CIGs, BPM, CDSSs, and Case-Based Reasoning (CBR) (see, e.g., in [114]). However, this led to a significant fragmentation of perspectives and disciplines. On the one hand, the high fragmentation fostered a rich set of methods and tools to deal with the issue. On the other hand, it may have had a negative impact on the concrete real-world applications that a unified and compelling vision could have been able to deliver. Probably, this is part of the reason why the results, in terms of concretely applied methods and tools (e.g., formalisms for representing CGs or software engines to parse and execute CIGs), remain limited compared to its to the potential.

More than a decade ago, a new discipline called Process Mining emerged, proposing techniques to mine processes starting from real process execution. It also provided generic techniques and methods to enrich existent processes and to measure the adherence of the data to a given process. Starting from that, due to the peculiarity of the clinical domain, a significant part of the community pushed to the creation of a new dedicated discipline, called PM4HC.

In coping with CGs, PM4HC can benefit from the aforementioned rich set of tools and methods, and can exploit them as a starting point for providing additional contribution. PM4HC needs to clearly position itself with respect to the theme of CGs, and identify the needs of the clinical domain to collect the relevant contribution of others disciplines in a unique *corpus*. In this paper, we provided an exhaustive description of the CGs domain from an healthcare perspective, and covered some of the main results and research direction from prior research.

**Figure 3:**
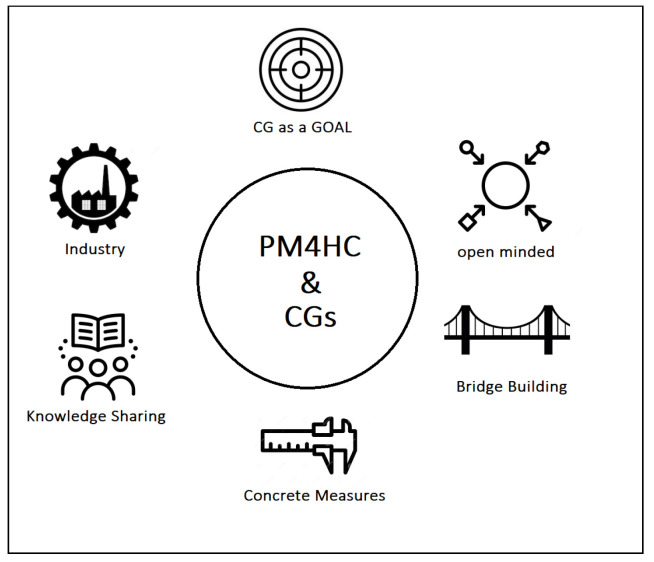
A general overview of some of the most relevant milestones in the modern age of Clinical Guidelines.

Looking ahead, thanks to the experience gained by interacting with physicians and healthcare providers in general, the authors are convinced that PM4HC can play a pivotal role in giving answers to many of the questions concerning CGs. In trying to figure out how to cope with these future challenges, we have identified a number of relevant points that summarize our position on this theme, in the near future. An overview is provided in Figure 3.

- **CG as a goal**. CGs represent an essential point in healthcare and PM4HC. Measuring process performance by considering real-world data is a primary aim of CC and CGs, and represents low-hanging fruits. The synergy with process discovery and process enhancement, in particular, open exciting scenarios about the potential of PM4HC, e.g., the former to potentially identify the most promising pattern of care given some successful clinical pathways, the latter to try to improve a given CGs with real-world data from a specific care unit.
- **Open-minded**. The fragmented approach in coping with CGs (CIGs, BPM, CBR, etc.) and the lack of communication among the members of these sub-communities caused the potential contribution of some technologies to be unexplored. The PM4HC community should be open-minded with regards to the results of other disciplines, and should be inclusive with respect to successful and promising methods which can be acquired and adapted. It is well positioned to play a unifying role for the broader field.
- **Bridge Building**. The cultural gap between computer scientists and healthcare providers is one of the main challenges, and distinguishing features, of PM4HC. This gap is mainly due to many years of specialization and can play a critical role in leading a joint project to success or failure. To make the communication more efficient, and increase the chances to come to successful results, physicians, nurses, caregivers, and administrative workers in healthcare should be invited to play an active role in all the steps of the projects and, more in general, also invited to contribute in terms of vision for the future of the discipline. Beside the clear communication-related issues of the cultural gap, there is also the remarkable aspect of sustainability. Bridging the gap between computer scientists and healthcare experts also encompasses the fact that proposed technological solutions must not become overwhelming with respect to the daily clinical workload. In other words, bridging the cultural gap also means to propose solutions able to fit with the actual working environment and that can provide clear benefits beside the additional effort that they may require (e.g., time spent for data entry, meetings, etc.).
- **Concrete measures**. In many cases, CGs presented in papers did not lead to concrete applications. Unfortunately, CGs still remain on paper in many hospitals. We think that being able to validate our future proposals with feedback from the domain experts, and providing concrete measures of the benefits generated by PM4HC solutions could convince physicians to adopt PM4HC tools. In this direction, shared methods to validate *on the field* future works and tools must be encouraged, when possible.
- **Knowledge sharing**. We strongly believe in teamwork and in the cooperation among research centers and healthcare institutions across different countries. To this end, initiatives aimed at sharing knowledge about community members working on CGs implementations should be promoted and supported.
- **Industry**. Proof of concepts or prototypes are the most common way for the scientific community to deliver their intellectual product. However, to make the jump from prototypes to commercial products which efficiently support daily clinical practice, the interaction with healthcare companies cannot be avoided and should be elicited as one of the aims of our community. The creation of a process-oriented culture with commercial vendors, in terms of data entry/presentation, ontologies, and data export should be pursued and joint project should be encouraged.

The above-mentioned points, and the CGs field as a whole, would significantly benefit by a comprehensive framework that incorporates knowledge governance, academic–industry partnership, and fast evidence production and assimilation. The design and development of such a framework is planned to be the next step of the authors and of the corresponding consortium. In doing that, the authors will take inspiration from initiatives such as Magic (http://magicproject.org/) [115] that developed frameworks for rapidly producing validate evidence and testing CDSS interventions.
